# Aicardi–Goutières syndrome protein TREX1 suppresses L1 and maintains genome integrity through exonuclease-independent ORF1p depletion

**DOI:** 10.1093/nar/gkx178

**Published:** 2017-03-15

**Authors:** Peng Li, Juan Du, John L. Goodier, Jingwei Hou, Jian Kang, Haig H. Kazazian, Ke Zhao, Xiao-Fang Yu

**Affiliations:** 1 Institute of Virology and AIDS Research, First Hospital of Jilin University, Changchun, Jilin 130061, China; 2 Bloomberg School of Public Health, Johns Hopkins University School of Medicine, Baltimore, MD 21205, USA; 3 Institute of Genetic Medicine, Johns Hopkins University School of Medicine, Baltimore, MD 21205, USA

## Abstract

Maintaining genome integrity is important for cells and damaged DNA triggers autoimmunity. Previous studies have reported that Three-prime repair exonuclease 1(TREX1), an endogenous DNA exonuclease, prevents immune activation by depleting damaged DNA, thus preventing the development of certain autoimmune diseases. Consistently, mutations in TREX1 are linked with autoimmune diseases such as systemic lupus erythematosus, Aicardi–Goutières syndrome (AGS) and familial chilblain lupus. However, TREX1 mutants competent for DNA exonuclease activity are also linked to AGS. Here, we report a nuclease-independent involvement of TREX1 in preventing the L1 retrotransposon-induced DNA damage response. TREX1 interacted with ORF1p and altered its intracellular localization. Furthermore, TREX1 triggered ORF1p depletion and reduced the L1-mediated nicking of genomic DNA. TREX1 mutants related to AGS were deficient in inducing ORF1p depletion and could not prevent L1-mediated DNA damage. Therefore, our findings not only reveal a new mechanism for TREX1-mediated L1 suppression and uncover a new function for TREX1 in protein destabilization, but they also suggest a novel mechanism for TREX1-mediated suppression of innate immune activation through maintaining genome integrity.

## INTRODUCTION

The genomic encoding of RNAs and proteins is essential for cells to survive. Conversely, damage to the genome leads to self-activation of the immune system, most commonly through the cGAS-STING-IRF3 pathway (1). Genome damage can be triggered by various conditions, such as oxidation (1) or ribonucleotide misincorporation (2–4). Therefore, cells utilize multiple endogenous factors to maintain the integrity of their genomic DNA and it is possible in theory that malfunctioning of these factors can lead to immune activation.

Endogenous retroelements are potential threats to genome integrity. The replication of retroelements involves a process called target-primed reverse transcription (TPRT) (5), which involves nicking of genomic DNA and sometimes leads to DNA breaks (6,7). Indeed, various retroelements, such as the autonomous long interspersed element type 1 (L1), are thought to be triggers of an innate immune activation that leads to the production of interferon (IFN) (8–12). In fact, L1 has been shown to induce IFN in human cell lines and a mouse model (13).

Three-prime repair exonuclease 1 (TREX1) is associated with autoimmune diseases such as Aicardi–Goutières syndrome (AGS, MIM: 225750) and familial chilblain lupus (FCL, MIM: 610448). These diseases share similar phenotypes and have been associated with abnormal immune activity triggered by DNA and/or RNA fragments that would normally be removed from cells (14,15). Consistently, TREX1 depletes DNA through its 3΄-to-5΄ DNA exonuclease activity and it acts on both single-stranded DNA (ssDNA) and double-stranded DNA (dsDNA) (16–18). Multiple studies have confirmed that TREX1 is a part of the host's immune system (1,14,19–22) and serves to prevent the self-activation of cGAS. However, the most significant finding is that knocking out *TREX1* leads to auto-inflammatory symptoms in mice (8,23,24).

Interestingly, multiple retroelements, including L1, are natural targets of TREX1. Indeed, elevated L1 ssDNA levels are observed in the heart tissue of *TREX1*^−/−^ mice (8). This phenomenon led to the assumption that exonuclease activity contributes to TREX1-mediated L1 suppression. However, although being associated with activation of autoimmunity and autosomal-dominant AGS (25), the TREX1 mutant D200N has been reported as exonuclease active (25) yet with no potency to suppress L1 retrotransposition (8). Thus, the role of TREX1 exonuclease activity remained controversial (25,26). These findings therefore raise the question of whether TREX1 might suppress L1s through an exonuclease-independent pathway. We now demonstrate that TREX1 can inhibit retrotransposition activity in a cell culture assay through loss of L1-encoded ORF1 protein. Revealing the exact mechanism that explains how TREX1 suppresses L1 activity could provide a greater depth of understanding of endogenous L1 regulation as well as the relationship between AGS-associated proteins, retroelement activity and cellular immune regulation.

## MATERIALS AND METHODS

Plasmid construction: the single-exon TREX1 gene was retrieved directly from the human genome by polymerase chain reaction (PCR) and then subcloned into VR1012 (containing a CMV promoter/enhancer, intronA, multiple cloning sites and BGH polyA signal sequence) (27,28) with a FLAG tag at the C-terminus. Various site mutations in TREX1 were introduced using standard site-directed mutagenesis techniques. All mutant constructs were sequence-confirmed. An EGFP gene was then inserted at the C-terminus of TREX1 to generate the plasmid expressing EGFP–TREX1 fusion protein. The SAMHD1-expressing vector (11), 99 PUR RPS EGFP (L1-RP) (29), 99 PUR JM111 EGFP (JM111) (29), pc-L1-1FH (L1-1FH) (30), pc-L1-2FH (L1-2FH) (31) and sL1-ORFeus-HS (sL1) (32) have been described. The construction of pc-L1-2FH was similar to that of L1-1FH, with ORF2p tagged with both FLAG and HA tags on the C-terminus (31). The ORF1 gene alone was amplified from L1-RP and inserted into VR1012 (vrORF1) and pmCherryC1 (pmORF1). The L1 5΄-UTR was amplified and inserted into pGL3-Basic to construct a 5΄-UTR-driven firefly luciferase-expressing vector (5UTR-Luc).

Four TREX1-specific shRNAs (shTREX1) with the following target sites were cloned in the lentiretroviral vector pLKO.1-puro (Addgene, Cambridge, MA, USA):

shTREX1-1, 5΄-AAGGTCACGGAGCTGTGCCTG-3΄;

shTREX1-2, 5΄-AACACGGCCCAAGGAAGAGCT-3΄;

shTREX1-3, 5΄-AAGACCATCTGCTGTCACAAC-3΄;

and shTREX1-4, 5΄-AAGGACCCTGGAGCCCTATCC-3΄.

Antibodies: the following antibodies were used to detect protein expression: anti-Calnexin from Proteintech (Chicago, IL, USA), anti-tubulin and anti-TREX1 from Abcam (Cambridge, MA, USA), anti-histone from GenScript (Piscataway, NJ, USA), anti-HA from Invitrogen (Carlsbad, CA, USA), anti-FLAG from Sigma (St. Louis, MO, USA) and anti-c-myc from Millipore (Billerica, MA, USA). The fluorescent AlexaFluor 488 goat anti-rabbit IgG was from Life Technologies (Eugene, OR, USA). All antibodies were used according to the manufacturers’ protocols.

Protein modeling: the structure of TREX1 has been previously published (PDB: 2OA8) (33). Modeling and color manipulation were performed with PyMOL software (http://www.pymol.org, version 1.6.9).

Cell culture: human embryonic kidney HEK293T and HeLa cells were grown in Dulbecco's Modified Eagle's Medium (DMEM) medium with 10% fetal bovine serum (FBS) (Hyclone), GlutaMax and Pen-strep (Invitrogen). No additional cell authentication or mycoplasma contamination testing was performed. All transfections used Lipofectamine 2000 (Invitrogen) reagent.

TREX1-specific shRNA transduction: for shRNA-based RNA silencing of TREX1, HEK293T cells were transfected with pMDLg/pRRE (Addgene), pRSV-Rev (Addgene), pHEF-VSVG (from Dr L.-J. Chang, through the AIDS Research and Reference Reagent Program, Division of AIDS, NIAID, NIH) and each of four TREX1-specific shRNAs. The cells were washed with fresh DMEM (supplemented with 10% FBS; Invitrogen) to remove free plasmids at 24 h post-transfection and the supernatant of the culture containing pseudotyped lentivirus packaging shTREX1 virions was collected after another 24 h. The pseudotyped lentivirus was then used to infect HeLa cells. The infected cells were selected with 5 μg/ml puromycin for 7 days beginning at 2 days post-infection. The knockdown potency of TREX1 was tested with western blotting.

Cytotoxicity test: the cytotoxicity test was performed with the TransDetect Cell Counting Kit (CCK, from Transgen). HEK293T cells seeded on 24-well plates were transfected with 450 ng of empty or TREX1-expressing vector (50, 150 and 450 ng dosage). At 24 h post-transfection, the cells were re-seeded onto a 96-well plate. After 48 h (the common time point for the test) or 96 h (the time point for the EGFP-based L1 assay) post-transfection, 10 μl of CCK reagent were added to each well and after a further 4 h, the absorption of the samples was examined at 450 nm with an iMarkMacroplate Reader (Bio-Rad, Hercules, CA, USA). DMEM medium (containing 10% FBS) was used as a negative control to remove the background noise.

Co-immunoprecipitation (co-IP): the co-IP experiments were performed as previously reported (34). HEK293T cells were first transfected with TREX1- and/or ORF1p-expressing vectors. The cells were then harvested at 48 h post-transfection, washed with 1× phosphate-buffered saline and suspended in lysis buffer (50 mM Tris–HCl [pH 7.5], 150 mM NaCl and 0.5% NP-40, supplemented with Roche protease inhibitor cocktail). Samples were sonicated at 15% power for 60 s with a 1 s break every 1 s and then centrifuged to obtain a clear supernatant. Input samples were incubated with a FLAG^®^ Immunoprecipitation Kit (Sigma) for 3 h, then washed several times with wash buffer (20 mM Tris-HCl [pH 7.5], 100 mM NaCl, 0.1 mM ethylenediaminetetraacetic acid (EDTA) and 0.05% Tween 20). The samples were then eluted with 100 mM glycine-HCl (pH 2.5) and used for subsequent experiments or frozen at −80°C.

Exonuclease assays: the exonuclease assay was performed as previously described (35). TREX1 and its mutants were first expressed in HEK293T cells, isolated through co-IP and subjected to western blotting to determine the protein levels of purified TREX1. Similar amounts of TREX1 were then used to digest linearized VR1012 in the reaction buffer (20 mM Tris–HCl [pH 7.5], 5 mM MgCl_2_, 2 mM dithiothreitol (DTT), 100 μg/ml bovine serum albumin) at 25°C for 20 min. The remaining DNA was examined by agarose electrophoresis.

L1 assays: the L1 retrotransposition assay has been previously described (11,29,36). In brief, L1 plasmid (i.e. retrotransposition-competent L1-RP or sL1, or retrotransposition-incompetent JM111) was transfected into HEK293T cells at 2 μg per well in 12-well plates, together with VR1012 or one of the test plasmids. The cells were selected by the addition of puromycin (final concentration, 5 μg/ml) at 48 h post-transfection. GFP-positive cells were examined 48 h later by flow cytometry using FACSCalibur. Gating exclusions were based on background fluorescence of the plasmid JM111, an L1 construct containing two point mutations in ORF1p that completely abolish retrotransposition (37); 20 000 single-cell events per sample were gated and analyzed using CellQuest Pro (version 5.2).

L1 neo assays: for the mneoI-based L1 retrotransposition assay, 2 μg LcRPS-mneoI (L1 neo) (38) were co-transfected into HeLa-HA cells (generous gift from Dr Astrid M. Roy-Engel from Tulane University) seeded on 12-well plates with 500 ng empty vector or the TREX1 expression vector (TREX1-WT or mutants: D130A, R114H, D200N). At 24 h post-transfection, transfected HeLa-HA cells were re-seeded into a T25 flask. And after another 3 days, the cells were selected with G418 (600 μg/ml, final concentration) for 13 days. Finally, the cells were fixed with 4% paraformaldehyde and colonies were stained with 0.4% Giemsa.

Protein rescue from proteasome-mediated degradation: the rescue of APOBEC3G from Vif-induced proteasomal degradation was performed as previously described (27,39), with minor variation. In brief, APOBEC3G and Vif-expressing vectors were co-transfected into HEK293T cells. At 18 h post-transfection, proteasome inhibitor MG132 was added to the cell culture medium at various final concentrations (1, 5 and 25 μM, respectively). The cells were collected after another 18 h and subjected to western blotting for detection of protein levels. A similar protocol was applied to rescue ORF1p from TREX1-mediated degradation.

Quantitative real-time reverse transcription PCR (qRT-PCR): the RNA from samples of interest was first extracted with an EasyPure RNA Kit (Transgen, Beijing, China) with DNase treatment as part of the extraction procedure and then subjected to reverse transcription with EasyScript First-Strand cDNA Synthesis SuperMix (Transgen). The qRT-PCR was performed with TransStart Top Green qPCR SuperMix (Transgen) and specific primers. The reactions were performed under the following conditions as suggested by the manufacturer: 94°C for 30 s, followed by 40 cycles of 94°C for 5 s and 60°C for 30 s, followed by a dissociation protocol. Single peaks in the melting curve analysis indicated specific amplicons. The primers used for qRT-PCR in this study are indicated in Supplementary Table S1.

L1s occupy about 17% of human genome. To exclude host genome from the qRT-PCR tests, the samples were thoroughly treated with DNase during RNA extraction. An RT- control (i.e. without reverse transcriptase) was prepared for each sample during the cDNA synthesis and used for a parallel qRT-PCR test to detect any possible contamination of genomic DNA. RT+ data were considered informative only if the cycle number of L1 was five lower than that of the associated RT- data, which means a >32-fold difference in cDNA levels, and should exclude genome contamination from final conclusion. The RT- data were not shown.

PCR: the PCR assay was performed to confirm TREX1's effect on the stability of full-length L1 RNA. In brief, L1-RP and TREX1-expressing vectors were co-transfected into HEK293T cells. At 48 h post-transfection, the RNA extraction and cDNA synthesis were performed as mentioned above. An RT- control was included during the cDNA synthesis, to rule out contamination by genomic or plasmid DNA. The PCR was performed with TransTaq DNA Polymerase High Fidelity (Transgen). The reactions were performed under the following conditions as suggested by the manufacturer: 94°C for 5 min, followed by 30 cycles of 94°C for 30 s, 55°C for 30 s and 72°C for 4 min, followed by 72°C for 10 min.

The primers used for PCR in this study were:

L1-3, forward (5΄-CAAACACCGCATATTCTCACTCA-3΄);

*EGFP*, forward (5΄-TGACCCTGAAGTTCATCTGC-3΄);

*GAPDH*, forward (5΄-GCAAATTCCATGGCACCGT-3΄) and reverse (5΄-TCGCCCCACTTGATTTTGG-3΄).

The amplicon based on L1-3 forward and EGFP forward covers regions of both the L1 and the EGFP cassette. The L1 part of this fragment ensures it represents the full-length L1 RNA transcribed from the 5-΄UTR instead of the intron-interrupted EGFP mRNA transcribed from the CMV promoter. The EGFP gene does not exist in the human genome, thus amplifying the EGFP part would help to exclude possible contamination from endogenous L1 DNA/RNA. Both primers of *GAPDH* target the same exon, thus were used not only as the loading control, but also to further exclude genome contamination during the PCR.

Cytoplasm retroelement L1 DNA measurement: 1 μg empty vector or 1 μg TREX1 expression vector (TREX1-WT or mutants: D130A, R114H, D200N) was transfected into HeLa cells seeded on 12-well plates. At 4 days post-transfection, transfected cells were harvested and processed for cytoplasmic nucleic acid separation. The cells were resuspended in 100 μl lysis buffer (20 mM HEPES/KOH [pH 7.6], 150 mM NaCl, 0.5 mM DTT, 0.5 mM Phenylmethanesulfonyl fluoride (PMSF)) and then 1 μl 2.5% digitonin solution (100×) was added, mixed gently and incubated at room temperature for 10 min. After centrifuging at 1000 x *g* for 5 min, supernatant was transferred to a new tube as the cytoplasmic material. The pellet contained nuclear materials and could be directly used to make samples for western blotting. DNA was extracted from the cytoplasmic material by the QIAamp DNA Mini Kit (from QIAGEN). Finally, equal volumes of cytoplasmic DNA were analyzed by L1-specific primers (L1-1, L1-3) using qRT-PCR.

Fluorescence imaging: pmORF1 and/or GFP-TREX1 were transfected into HEK293T cells. At 24 h post-transfection, the cells were subjected to live cell imaging, or fixed with 4% paraformaldehyde and stained with anti-calnexin antibody, followed by fluorescent AlexaFluor 488 goat anti-rabbit IgG. The fluorescence was then examined with an Olympus IX51 inverted microscopy system and photographed with a Pooher PDC50-C CCD camera (Pooher, Shanghai, China).

Luciferase assays: the Dual-Luciferase Reporter Assay System from Promega (Fitchburg, WI, USA) was used to detect whether TREX1 or SAMHD1 affected the promoter activity of the LINE-1 5΄-UTR and CMV promoter. In brief, the 5΄-UTR and CMV promoters were amplified with PCR and subcloned into pGL3 (containing the firefly luciferase gene) to generate pGL3-5UTR and pGL3-CMV. The Renilla luciferase vector contained an SV40 promoter. HEK293T cells were then co-transfected with pGL3/pGL3-5UTR/pGL3-CMV, the Renilla luciferase vector and mock/VR1012/TREX1/SAMHD1. At 48 h post-transfection, the cells were lysed and treated according to the manufacturer's protocol for luciferase detection. Readings of pGL3 were used to remove the background noise and are not shown.

Comet assays: the comet assay was conducted according to published procedures (35). In brief, HeLa cells were transfected with pc-L1-1FH, along with the vector expressing wild-type TREX1 or one of its AGS-associated mutants, or the control vector. At 96 h post-transfection, the cells were mixed with 0.5% low-melting temperature agarose (Agarose LMP, Gen-view Scientific Inc., FL, USA) at 37°C, placed on a precleaned microscope slide that was already covered with a second layer of 0.5% normal melting agarose (Regular Agarose G-10, Biowest, Spain) and immediately covered with a coverglass and kept at 4°C for 5 min. After the cover glass was gently removed, the slide was covered with a third layer of low-melting agarose by using another cover glass, then horizontally placed at 4°C. The solidified slide was then immersed in a lysing solution (1% sodium sarcosinate, 2.5 M NaCl, 100 mM Na_2_-EDTA, 10 mM Tris [pH 10.0] and 1% freshly added Triton X-100) for 1 h to release and unfold the DNA, followed by the electrophoretic buffer (1 mM Na_2_-EDTA and 300 mM NaOH) for 20 min to unwind the DNA. The electrophoresis was then conducted for another 20 min at 25 V. The slide was then horizontally immersed in 0.4 M Tris–HCl (pH 7.5) and stained with 4΄,6-diamidino-2-phenylindole (DAPI) (Sigma) for 5 min before observation under the Olympus IX51 inverted microscopy system. The tail moment of comets was measured for 100 cells for each sample using Comet Assay IV software.

## RESULTS

### AGS-associated exonuclease-active mutants of TREX1 fail to suppress L1

**Figure 1.**
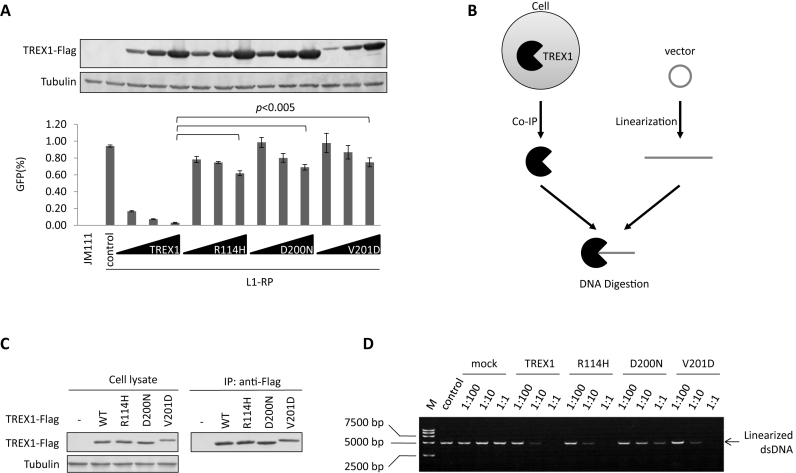
AGS-associated exonuclease-active mutants of TREX1 do not suppress L1. (**A**) L1 assay results indicating that AGS-associated mutations significantly compromise TREX1's potency against L1 activity. Vectors expressing wild-type TREX1 or its AGS mutants in a dose manner (50, 150 and 450 ng) were co-transfected with 2 μg L1-RP into HEK293T cells seeded on a 12-well plate to examine the possible potency against L1 retrotransposition. At 4 days post-transfection, EGFP-positive cells were determined by flow cytometry. JM111 was used as the negative control for flow cytometry gating and VR1012 was the empty vector used as the negative control for TREX1 expression. The western blotting results (above) show the expressed levels of TREX1 and its mutants. (**B**) Cartoon showing the analysis of TREX1-mediated DNA digestion. FLAG-tagged TREX1 was expressed in HEK293T cells and purified through affinity chromatography. The target vector was first linearized then incubated with purified TREX1 protein for 20 min. The mixture was subjected to agarose electrophoresis for DNA detection. (**C**) Western blotting results showing the protein levels of TREX1 in both cell lysates and eluates in co-immunoprecipitation (co-IP). Three micrograms of vectors expressing TREX1 or its AGS mutants were transfected into HEK293T cells seeded on a 6-cm dish. At 48 h post-transfection, the cells were harvested for the IP assay shown in C and the DNase activity assay shown in D. (**D**) Agarose electrophoresis results showing that AGS-associated mutants R114H and V201D, but not D200N, can efficiently digest linearized DNA. M, DL15 000 DNA marker (Takara). Vector VR1012 was linearized and used as the plasmid substrate. The ratios shown are dilution rates of extract TREX1 protein. All the data shown in this figure are representative of at least three independent experiments. The error bars shown in A indicate the SD of three replicates within one experiment.

Previously, Stetson *et al*. (8) tested three AGS-associated TREX1 mutants, R114H, D200N and V201D, for their potency in regulating retrotransposition. Using a widely used EGFP-based L1 retrotransposition assay (Supplementary Figure S1) (29), we also observed that all three of these TREX1 mutants were defective in TREX1-mediated L1 suppression (Figure 1A). The suppressive effect of TREX1 was also confirmed in a neomycin-based L1 assay (Supplementary Figure S2). To confirm the DNA exonuclease activity of TREX1 D200N, we extracted tagged TREX1 protein from eukaryotic HEK293T cells by immunoprecipitation (Figure 1B), rather than purifying it from a prokaryotic expression system as was done in previous studies (25,26,40,41). We reasoned that by doing so, the TREX1 protein would interact with its natural partners expressed in human cells and thus should reflect the *in vivo* activity of TREX1. We then tested its DNA exonuclease activity by digestion of a linearized plasmid (Figure 1B). We found that the AGS-associated TREX1 mutation D200N was both inactive for L1 suppression and significantly compromised in digesting DNA (Figure 1C and D), confirming the findings of Lehtinen *et al*. (26), but in disagreement with those of Rice *et al*. (25). Significantly, however, the other two AGS-associated mutations, R114H and V201D exhibited a potency for DNA degradation that was similar to that of wild-type TREX1 (Figure 1C and D), despite their weakened capacity with regard to L1 regulation (Figure 1A). These results suggested that the DNA exonuclease might not be essential for TREX1-mediated L1 suppression.

### Exonuclease-inactive TREX1 mutants potently suppress L1 activity

**Figure 2.**
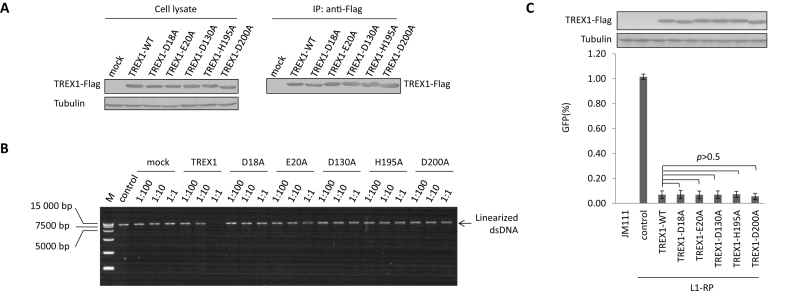
An exonuclease-independent mechanism contributes to TREX1-mediated L1 suppression. (**A**) Western blotting results showing the protein levels of TREX1 in both cell lysates and co-IP eluates. Three micrograms of vectors expressing TREX1 or its DNase-defective mutants were transfected into HEK293T cells seeded on a 6-cm dish. At 48 h post-transfection, the cells were harvested for the IP assay shown in A and DNase activity assay shown in B. (**B**) Agarose electrophoresis results showing that wild-type TREX1, and not its exonuclease-deactivated mutants, can digest linearized DNA. M, DL15 000 DNA marker (Takara). Linearized L1-RP was used in place of VR1012 as the substrate to confirm that the tested mutants did not affect the L1 assay results by compromising exonuclease activity. The ratios shown are dilution rates of extract TREX1 proteins. (**C**) L1 assay results showing that exonuclease-deactivated TREX1 mutants maintain potency against L1 replication. A total of 450 ng of VR1012 empty vector or vectors expressing TREX1 or its DNase-defective mutants were co-transfected with 2 μg L1-RP into HEK293T cells seeded on a 12-well plate to examine potency against L1 retrotransposition. At 4 days post-transfection, EGFP-positive cells were determined by flow cytometry. JM111 was used as the negative control for flow cytometry gating, and VR1012 was the empty vector used as the negative control for TREX1 expression. The western blotting results show the expressed levels of TREX1 and its mutants. All the data shown in this figure are representative of at least three independent experiments. The error bars shown in C indicate the SD of three replicates within one experiment.

To verify this hypothesis, we introduced various mutations into TREX1 at positions that have previously been predicted to be critical for the protein's exonuclease activity (Supplementary Figure S3) (33). DNA digestion assays confirmed that mutation of these critical sites at least compromised, if not abolished, the exonuclease activity of TREX1 (Figure 2A and B). However, with expression levels similar to wild-type TREX1, these exonuclease-inactive mutants were fully functional in regulating L1 activity (Figure 2C), which was also confirmed in a neomycin-based L1 assay (Supplementary Figure S2). Thus, combined with the data above (Figure 1), these results confirm that DNA exonuclease activity may not be essential for TREX1-mediated L1 suppression; an additional mechanism(s) may also contribute.

Recent studies have suggested that TREX1 suppresses the activation of the endogenous DNA sensor cGAS and prevents autoimmunity by reducing cytosolic DNA (42,43), especially retroelement DNA (8). Our findings above suggest that both DNA digestion and an additional mechanism may affect cytosolic levels of L1 DNA. Thus, we quantified the relative levels of cytosolic L1 DNA in HeLa cells expressing exogenous TREX1 or its mutants (D130A that fails in DNA digestion (Figure 2B) but not L1 suppression (Figure 2C), R114H that fails in L1 suppression (Figure 1A) but not digestion (Figure 1D) or D200N that is attenuated in both (Figure 1A and D). Compared with wild-type TREX1, D130A and R114H that fail in DNA digestion and L1 suppression, respectively, were both less competent in reducing cytoplasmic levels of L1 DNA (Supplementary Figure S4). D200N that fails in both functions of TREX1 was almost completely impotent in regulating cytosolic L1 DNA levels (Supplementary Figure S4). These data confirm our hypothesis that TREX-mediated DNA digestion and L1 suppression can be uncoupled.

### TREX1 suppresses L1 activity and L1-induced genome nicking by reducing L1 ORF1p levels

**Figure 3.**
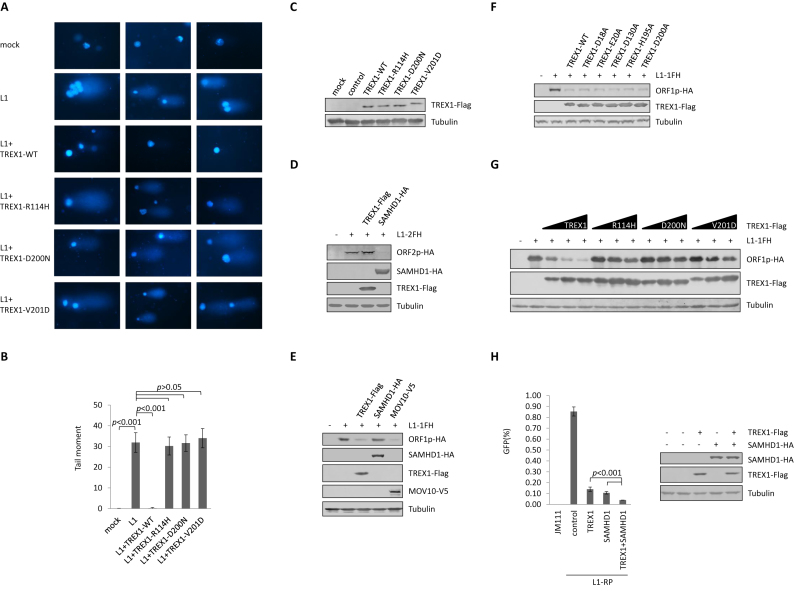
TREX1 suppresses L1 activity and L1-induced genome nicking by reducing L1 ORF1p levels. (**A**) Comet assay results concerning TREX1 suppression of L1-induced genome damage. HeLa cells were transfected with L1-1FH (2 μg), along with 500 ng control vector or the vector expressing TREX1 or one of its mutants on a 12-well plate. The cells were then subjected to the comet assay and fluorescent imaging at 96 h post-transfection. The data are representative of three independent experiments and three random areas are shown for each sample from the same experiment. (**B**) The tail moment of the comets in A was analyzed for 100 cells for each sample using Comet Assay IV software. (**C**) Western blotting results indicating corresponding levels of TREX1 in each sample shown in A. (**D**) Western blotting results showing that TREX1 does not reduce the protein levels of ORF2p. TREX1 (500 ng) or SAMHD1 (500 ng) expression plasmids were co-transfected with L1-2FH (2 μg) into HEK293T cells seeded on a 12-well plate. At 48 h post transfection, the cells were harvested for western blotting. SAMHD1 was previously reported to reduce ORF2p and is shown as a positive control. (**E**) Western blotting results showing that TREX1 potently reduces protein levels of ORF1p. TREX1 (500 ng) or SAMHD1 (500 ng) or MOV10 (500 ng) expression plasmids were co-transfected with L1-1FH (300 ng) into HEK293T cells seeded on a 12-well plate. At 48 h post -transfection, the cells were harvested for western blotting. SAMHD1 was introduced as a negative control, and MOV10 as a positive control (30,59). (**F**) Western blotting results showing that exonuclease-deactivated TREX1 mutants maintain the ability to reduce ORF1p levels. Five hundred nanogram TREX1 WT or DNase mutant expression plasmids were co-transfected with L1-1FH (300 ng) into HEK293T cells seeded on a 12-well plate. At 48 h post transfection, the cells were harvested for western blotting. (**G**) Western blotting results showing that AGS-associated mutations compromise TREX1's ability to deplete ORF1p. Vectors expressing TREX1 or its AGS mutants in a dose manner (50, 150 and 450 ng) were co-transfected with L1-1FH (300 ng) into HEK293T cells seeded on a 12-well plate. At 48 h post transfection, the cells were harvested for western blotting. (**H**) L1 assay results indicating that the suppressive effects of TREX1 and SAMHD1 against L1 replication are additive. Vectors expressing TREX1 (500 ng) or SAMHD1 (500 ng) or both plasmids were co-transfected with 2 μg L1-RP into HEK293T cells seeded on a 12-well plate to examine possible potency against L1 retrotransposition. The western blotting results show the expressed levels of TREX1 and SAMHD1. All the data shown in this figure are representative of at least three independent experiments. Three random areas are shown for each sample from the same experiment in A. The error bars shown in B indicate the standard error of the mean (SEM) of three independent experiments, and those in H indicate the SD of three replicates within one experiment.

TPRT is essential for L1 replication, which involves genome nicking and sometimes introduces damage to genomic DNA (6,7). L1-triggered genome damage results in a tail-like migration pattern of genomic DNA in the comet assay (35), as previously reported (7,44) and as we confirmed here (Figure 3A and B). Wild-type TREX1 efficiently protected the genome from L1-triggered damage (Figure 3A, B and C), also suggesting that TREX1 suppresses L1 activity at a step prior to L1-induced genome nicking. It is noteworthy that TREX1 mutants that are associated with AGS were compromised in suppressing L1-induced genome damage (Figure 3A, B and C).

We previously determined that SAMHD1, another AGS-associated protein, suppresses L1 activity by reducing the level of L1 ORF2p, which is critical for L1 replication (11). Unlike SAMHD1, TREX1 did not affect the protein levels of tagged ORF2p expressed from a full-length L1 (pc-L1-2FH (31)) (Figure 3D), despite ORF2p having endonuclease activity that is directly involved in L1-induced genome nicking (45). Interestingly, however, TREX1 potently reduced the level of tagged ORF1p expressed from the full-length L1 construct pc-L1-1FH (Figure 3E). L1-encoded ORF1p is also essential for L1 retrotransposition (46). Consistent with the phenomenon observed for L1 retrotransposition suppression (Figure 2C), exonuclease-inactive TREX1 mutants retained potency in reducing ORF1p in a dose-dependent manner (Figure 3F; Supplementary Figures S5A and B), as well as suppressing L1-induced genome nicking (Supplementary Figure S6). On the other hand, AGS-associated TREX1 mutants were defective in ORF1p removal (Figure 3G), a finding that also correlated with their potency against L1 activity (Figure 1A). Thus, multiple data indicate that TREX1 inhibits L1 retrotransposition and protects genome integrity through a depletion of L1 ORF1p. They also suggest that AGS-associated proteins TREX1 and SAMHD1 suppress L1 through different mechanisms, which are apparently additive (Figure 3H).

Cytotoxity was examined for two time points: at 48 h post-transfection, which is a common time point for the test; and at 96 h post-transfection, which is the time point for the EGFP-based L1 assay. These tests indicated that ORF1p reduction was not a result of TREX1-mediated induction of cytotoxicity in HEK293T cells (Supplementary Figure S7A). Also, to determine whether TREX1 reduces ORF1p by affecting the L1 promoter, we co-transfected TREX1-expressing vector and 5UTR-Luc (a firefly luciferase-expressing vector driven by the L1 5΄-UTR) into HEK293T cells, and tested cells for luciferase activity at 48 h post-transfection. TREX1 did not reduce ORF1p by affecting the promoter potency of the L1 5΄-UTR (Supplementary Figure S7B). Also, since a recent discovery reported that TREX1 also acts as an exoribonuclease (47), we transfected TREX1-expressing vector into HEK293T cells and then tested the levels of endogenous L1 RNA. The results suggested that TREX1 did not destabilize L1 RNA (Supplementary Figure S7C). To further verify that TREX1 did not destabilize full-length L1 and exclude the possible contamination of L1 fragments embedded within other genes, we transfected HEK293T cells with TREX1 and L1-RP, and ∼2 kb fragment of L1-RP mRNA was amplified (Supplementary Figure S7D). As a result, TREX1 expressed in different dosages did not affect the levels of this 2 kb fragment (Supplementary Figure S7E and F). Since the fragment covers the end of *ORF2*, 3΄-UTR and a major part of the EGFP cassette, it could only be transcribed together with the full-length L1 RNA from L1-RP. Thus, combined with our data with endogenous L1 RNA, we believe that TREX1 did not alter the stability of full-length L1 RNA. In addition, TREX1 reduced the levels of ORF1p in a plasmid containing only the ORF1 gene instead of full-length L1 sequence (Supplementary Figure S7G). To further confirm that TREX1 suppresses the L1 by targeting the ORF1p protein instead of a specific nucleotide sequence, we made use of sL1, which is a human synthetic L1 construct with greatly altered DNA sequence that still expresses wild-type ORF1p and ORF2p as amino acid sequences (Supplementary Figure S8A). Consistent with our hypothesis, TREX1 suppressed L1 and sL1 retrotransposition with similar potency (Supplementary Figure S8B), replicating our previous findings regarding SAMHD1 targeting of ORF2p (11).

**Figure 4.**
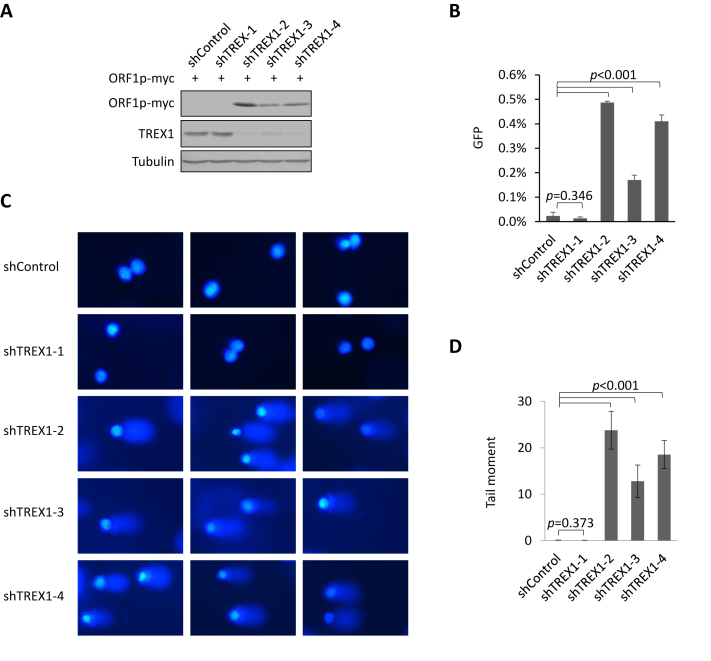
Endogenous TREX1 destabilizes ORF1p, suppresses L1 activity and protects genomic integrity. (**A**) Western blotting results showing that the levels of ORF1p protein are increased in HeLa cells in which endogenous TREX1 has been knocked down. ORF1p expression plasmid (300 ng) was transfected into five HeLa cell lines (HeLa shControl and four HeLa shTREX1 cells) seeded on a 12-well plate. At 48 h post-transfection, the cells were harvested for western blotting. (**B**) L1 assay results indicating that reducing endogenous TREX1 levels in HeLa cells results in an increase in L1-RP activity. Two micrograms of L1–RP were transfected into HeLa cells with shControl or shTREX1 constructs to examine the possible potency of L1 retrotransposition. (**C**) Results from comet assays suggesting that endogenous TREX1 protects genomic integrity. (**D**) The tail moment of the comets in C were analyzed for 100 cells for each sample using Comet assay IV software. All the data shown in this figure are representative of three independent experiments. Three random areas are shown for each sample from the same experiment in C. The error bars shown in B indicate the S\D of three replicates within one experiment, and those in D indicate the SEM of three independent experiments.

TREX1 is potently expressed in HeLa cells but not in HEK293T cells (Supplementary Figure S9). To test the role of endogenous TREX1 in L1 replication, we introduced four TREX1-specific shRNAs into HeLa cells and three of them led to a reduction in TREX1 expression to different degrees (Figure 4A). In the presence of TREX1 knockdown, exogenous ORF1p expression was elevated (Figure 4A) and L1-RP retrotransposition activity was significantly increased (Figure 4B). Consistently, results from the comet assay also showed that the integrity of the genomic DNA was compromised when the levels of endogenous TREX1 were reduced (Figure 4C and D). Therefore, endogenous TREX1 indeed regulates L1 retrotransposition and protects the genome from L1-induced damage.

### The TREX1 interaction is important but not sufficient for ORF1p depletion

**Figure 5.**
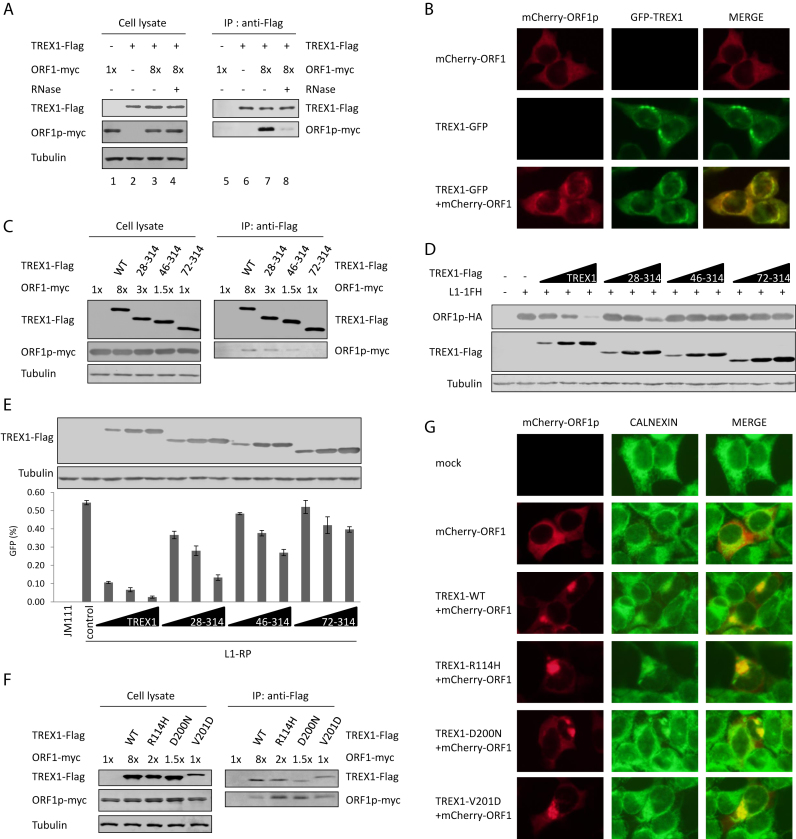
The TREX1–ORF1p interaction is important but not sufficient for TREX1-mediated depletion of ORF1p. (**A**) Co-IP results indicating that TREX1 interacts with ORF1p in an RNA-dependent manner. ORF1p (600 ng was 1×) and/or TREX1 (2 μg) expression plasmids were transfected into HEK293T cells seeded on 6-cm dishes. The transfection dose of the ORF1p-expressing vector was 8-fold higher than that for TREX1 (lanes 3 and 4) in order to achieve similar protein expression levels (lane 2). (**B**) Fluorescence imaging results showing the co-localization of TREX1 (green) and ORF1p (red) in live cells. TREX1-GFP (1 μg) and/or mCherry-ORF1 (1 μg) expression plasmids were transfected into HEK293T cells seeded on a 6-well plate. (**C**) Co-IP results showing that N-terminal truncations compromise TREX1's ability to interact with ORF1p. Two micrograms of vectors expressing TREX1 or its N-terminal truncations were co-transfected with the indicated amount of ORF1p (600 ng was 1×) expression plasmids into HEK293T cells seeded on 6-cm dishes. (**D**) Western blotting results indicating that N-terminal truncations compromise TREX1's ability to deplete ORF1p. Vectors expressing TREX1 or its N-terminal truncations in different doses (50, 150 and 450 ng) were co-transfected with L1-1FH (300 ng) into HEK293T cells seeded on a 12-well plate. At 48 h post transfection, the cells were harvested for western blotting. (**E**) L1 assay results indicating that N-terminal truncations compromise TREX1's ability to suppress L1. Vectors expressing TREX1 or its N-terminal truncations in different doses (50, 150, and 450 ng) were co-transfected with 2 μg L1-RP into HEK293T cells seeded on a 12-well plate to examine the possible potency of L1 retrotransposition. At 4 days post-transfection, EGFP-positive cells were determined by flow cytometry. JM111 was used as the negative control for flow cytometry gating and VR1012 was the empty vector used as the negative control for TREX1 expression. The western blotting results show the expressed levels of TREX1 and its mutants. (**F**) Co-IP results showing that AGS-associated TREX1 mutants maintain the ability to interact with ORF1p. Various doses of the ORF1p expression vector were used to achieve similar expression in the presence of different TREX1 mutants. Two micrograms of vectors expressing TREX1 or its AGS mutants were co-transfected with the indicated amount of ORF1p (600 ng was 1×) expression plasmids into HEK293T cells seeded on 6-cm dishes. (**G**) Fluorescence imaging results show that AGS-associated mutants still alter the subcellular localization of ORF1p (red); calnexin (green) is a protein marker labeling the endoplasmic reticulum (ER) (8). One microgram of VR1012 vector or vectors expressing TREX1 or its AGS mutants were co-transfected with 1 μg mCherry-ORF1 expression plasmid into HEK293T cells seeded on a 6-well plate. All the data shown in this figure are representative of at least three independent experiments. The error bars shown in E indicate the SD of three replicates within one experiment.

The data above suggested that TREX1 might induce the depletion of ORF1p at the protein level. Indeed, co-IP experiments indicated that TREX1 interacted with ORF1p and the interaction was RNA-dependent (Figure 5A). Live cell imaging also demonstrated the co-localization of TREX1 and ORF1p (Figure 5B), supporting the interaction *in vivo*. N-terminal truncations compromised TREX1's capacity to interact with ORF1p (Figure 5C). Consequently, these N-terminal truncation mutants showed a weakened ability to induce ORF1p depletion (Figure 5D). These mutants were also defective in L1 retrotransposition suppression (Figure 5E). However, the TREX1–ORF1p interaction alone was not sufficient for TREX1-induced ORF1p depletion, because AGS-associated TREX1 mutants that were defective in mediating ORF1p depletion (Figure 3G) maintained their binding ability for ORF1p (Figure 5F).

It was noteworthy that the cellular distribution of ORF1p was altered in the presence of TREX1 (Figure 5B). TREX1 was previously shown to localize to the endoplasmic reticulum (ER) (8,48). Fluorescent imaging indicated that fixation of cells with paraformaldehyde did not alter the localization of ORF1p or TREX1 or their co-localization (Supplementary Figure S10). Under the same conditions, ER attachment was observed for ORF1p in the presence, but not in the absence of TREX1 (Figure 5G), validating the interaction between TREX1 and ORF1p. However, ER attachment was apparently insufficient for TREX1-mediated depletion of ORF1p because AGS-associated TREX1 mutants were also located to the ER and maintained the co-localization with ORF1p (Figure 5G).

### Proteasome-mediated proteolysis contributes to the TREX1-induced depletion of ORF1p

**Figure 6.**
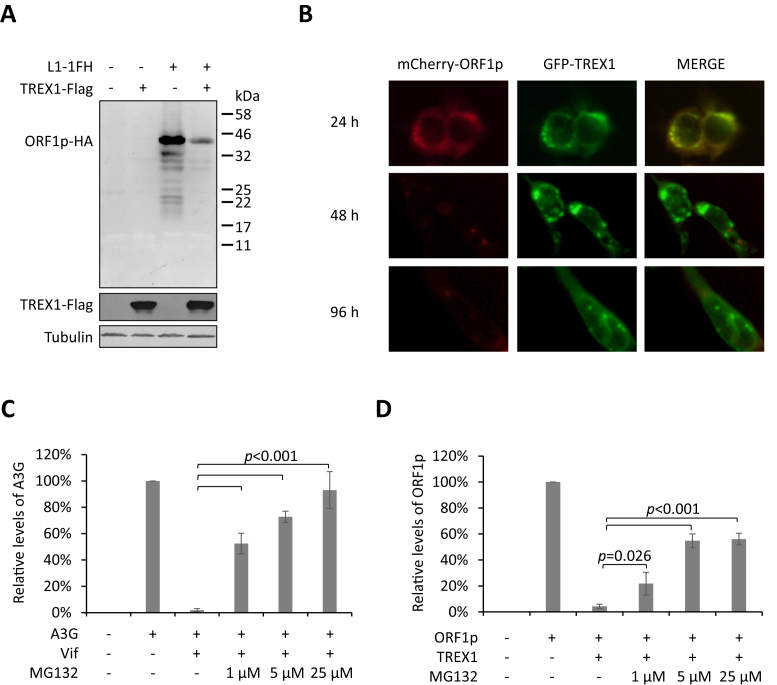
Proteasome proteolysis is involved in TREX1-mediated removal of ORF1p. (**A**) Full-length western blot showing that no smaller fragments of ORF1p are increased in concentration in the presence of TREX1. TREX1 (500 ng) and/or L1-1FH (300 ng) expression plasmids were transfected into HEK293T cells seeded on a 12-well plate. (**B**) Fluorescence imaging results for live cells showing that TREX1-induced ORF1p depletion occurs after a 24 h delay. HEK293T cells were co-transfected with ORF1p-mCherry and TREX1-EGFP expression vectors and subjected to fluorescence imaging at 24, 48 and 96 h post-transfection. TREX1-GFP (1 μg) and/or mCherry-ORF1 (1 μg) expression plasmids were transfected into HEK293T cells seeded on a 6-well plate. (**C**) Bar graph showing that proteasome inhibitor MG132 potently rescues APOBEC3G from Vif-induced degradation. VR1012 (900 ng) empty vector or Vif (900 ng) expression plasmid was co-transfected with A3G (300 ng) into HEK293T cells seeded on a 12-well plate. (**D**) Bar chart indicating that proteasome inhibitor MG132 rescues ORF1p from TREX1-mediated depletion. VR1012 (500 ng) empty vector or TREX1 (500 ng) expression plasmid was co-transfected with L1-1FH (300 ng) into HEK293T cells seeded on a 12-well plate. Data shown in A and B are representative of at least three independent experiments. Protein levels shown in B and D were quantified with ImageJ software and normalized to the levels of tubulin (as the loading control). Mean (± SE) values from three independent tests are shown.

Western blotting results indicated that no truncated fragment of ORF1p could be detected in the presence of TREX1 (Figure 6A), suggesting that a protein degradation process was involved in TREX1-mediated ORF1p reduction, rather than endopeptidase cleavage. Interestingly, when TREX1 and ORF1p were co-expressed, the co-localization of the two proteins was readily detected at 24 h post-transfection, but a more profound ORF1p depletion was observed after another 24 h (Figure 6B). This observed delay in TREX1-mediated ORF1p reduction suggests that TREX1 may not destabilize ORF1p directly, but instead may present ORF1p to some other cellular machinery for degradation, which takes time. A logical candidate for this degradation is proteasome-mediated proteolysis, which contributes to major pathways of protein degradation in eukaryotes (49).

Proteasome inhibitors were used to ascertain whether proteasomal proteolysis is involved in TREX1-mediated ORF1p depletion. Our data confirmed the potency of MG132 in suppressing Vif-induced proteasomal degradation of APOBEC3G (Figure 6C), as in our previous studies (27,39) (Supplementary Figure S11A). Repeated tests confirmed that MG132 rescued ORF1p expression from TREX1-mediated depletion (Figure 6D and Supplementary Figure S11B), indicating the likely involvement of proteasome-dependent protein degradation.

## DISCUSSION

The 3΄-to-5΄ exonuclease activity of TREX1 (16), the generation of cDNA by reverse transcription during retrotransposition (50), and especially the increase in retroelement ssDNA in the heart tissue of TREX1^−/−^ mice (8), led to the hypothesis that TREX1 suppresses retrotransposition by digesting reverse-transcribed cDNA. In the present study, however, we have found that TREX1-mediated L1 suppression can be exonuclease-independent. Indeed, multiple TREX1 mutants that are defective in digesting DNA share a similar potency against L1 when compared to wild-type TREX1. On the other hand, the AGS-associated mutants R114H and V201D maintained their exonuclease activity but were unable to efficiently suppress L1 replication. Further investigation indicated that the specific reduction of L1 ORF1p, but not ORF2p, contributes significantly to TREX1-mediated L1 suppression. This finding was verified by the failure of the AGS-associated TREX1 mutants to reduce ORF1p and inhibit L1. ORF1p is essential for the assembly of L1 ribonucleoprotein particles (RNPs) (31,51), which then enter into the nucleus, induce genome nicking (7,52) and subsequently synthesize L1 cDNA. Thus, by removing ORF1p, TREX1 could suppress L1 through an alternative mechanism operating several steps before retroelement cDNA synthesis.

Recent studies have indicated that TREX1 prevents immune activation by suppressing activation of the cGAS-STING pathway (42,53,54). Digested ssDNA/dsDNA released from the nucleus should contribute to TREX1-mediated immune suppression. Although we have determined that the AGS-associated TREX1 mutants R114H and V201D are exonuclease-active, our study does not deny the relationship between TREX1's exonuclease activity and cGAS/STING activation. In fact, coincident with reducing the levels of L1 ORF1p protein, TREX1 also suppressed L1-induced genome nicking, as shown by the comet assay, suggesting a novel way for TREX1 to prevent autoimmunity. It has been reported that genome damage can trigger immune activation (1), although the exact DNA sensor or pathway remains unclear. It is also noteworthy that AGS-associated proteins such as RNaseH2 (3,4,55,56), SAMHD1 (57) and TREX1 all protect genome integrity through multiple pathways. On the other hand, AGS-associated TREX1 mutants failed to suppress L1 or protect the genome from L1-induced DNA damage, despite the fact that some of these mutants are exonuclease-active. This evidence strongly indicates a potential contribution of genome damage to immune activation and the development of AGS.

It appears that a TREX1-mediated reduction in ORF1p contributes significantly to L1 suppression. The AGS-associated TREX1 mutants R114H and V201D maintain exonuclease activity but are defective in ORF1p depletion. Consequently, both mutants were significantly reduced in the ability to suppress L1. It is reasonable that TREX1 favors a suppression mechanism that occurs prior to L1 DNA synthesis, the point at which TREX1's exonuclease has an effect. Normally, TREX1 is mainly localized to the ER, instead of within the nucleus where retrotransposition occurs. Thus, TREX1's subcellular localization in theory determines that it may be unable to digest the L1 cDNA that is being reverse-transcribed and attached to the genome, but it could suppress L1 to a significant degree in the cytoplasm. This scenario, however, requires a mechanism different from cDNA digestion.

AGS-associated TREX1 mutants fail to suppress L1 activity, which should increase the synthesis of L1 cDNA. These elevated L1 cDNAs may subsequently saturate the capacity of TREX1 for DNA removal, which in turn could result in an overall increase in ssDNA in the cytoplasm. Therefore, the failure to suppress L1 replication and L1-induced genome damage provides a possible explanation for the ability of the exonuclease-active TREX1 mutants R114H and V201D to cause AGS. Attaching to perinuclear ER, on the other hand, most likely sets up the exonuclease TREX1 as a guardian, to prevent the release of nuclear DNA (including L1 cDNA) and the subsequent activation of cytosolic DNA sensors. Thus, TREX1 reduces cytosolic levels of L1 cDNA through at least two pathways: L1 ORF1p suppression and L1 cDNA digestion, a finding that is consistent with the reported phenomenon that retroelement cDNA is significantly increased in TREX1 knockout mice (8).

It is surprising to observe TREX1-mediated protein reduction, a novel function that, to our knowledge, has not been reported previously. Importantly, despite the recently discovered ribonuclease activity of TREX1 (47), we present evidence that TREX1 reduces ORF1p through a post-translational mechanism. Indeed, blocking proteasome proteolysis with the widely used inhibitor MG132 (27,34) rescued ORF1p in the presence of TREX1, suggesting that proteasome-mediated protein degradation is involved in TREX1-mediated depletion of ORF1p. Interestingly, although a TREX1–ORF1p protein interaction has been demonstrated, the interaction by itself is insufficient for ORF1p removal. Apparently an additional factor(s) is required. TREX1 localizes to the ER (8,48), where the proteasome is involved in ER-associated degradation of proteins (58). Interestingly, another endogenous complex termed SET is also attached to ER, and TREX1 has been reported as a component in SET (48). Yet, the re-localization of ORF1p onto the ER by TREX1 is not sufficient for TREX1-mediated ORF1p depletion, as tested with AGS-associated TREX1 mutants. Therefore, it seems that SET may not be the critical cellular partner of TREX1-induced ORF1p degradation or TREX1-mediated L1 suppression. Anyway, although many questions remain concerning TREX1 function, future studies on TREX1-mediated protein degradation will certainly benefit from the expanded understanding of the biological functions of TREX1 demonstrated here.

## Supplementary Material

Supplementary Data
