# Supplementary material for: Aicardi–Goutières syndrome protein TREX1 suppresses L1 and maintains genome integrity through exonuclease-independent ORF1p depletion
Source: Nucleic Acids Res. 2017 Mar 15;45(8):4619–31. doi: 10.1093/nar/gkx178 (PMC5416883; doi:10.1093/nar/gkx178)
Supplement: Supplementary Data [file gkx178_Supp.pdf]

Supplementary Figure legends

Supplementary Figure S1. Cartoon explaining the principle of the L1 assay. In the retrotransposition-competent vector L1-RP, an antisense EGFP expression cassette, driven by CMV promoter, is inserted into the 3'-UTR region of a sense L1 sequence. In addition, the EGFP gene is interrupted by an antisense gamma-globin intron. L1-RP is transcribed from its 5'-UTR. RNA splicing must occur to remove the intron. The spliced RNA is then reverse-transcribed and integrated into genomic DNA. Only after that can the EGFP signal be generated from the integrated CMV-EGFP cassette. Any direct transcription from the EGFP reporter cassette CMV promoter prior to insertion will not result in EGFP production because of the presence of the intron. The regions for L1-specific qRT-PCR primer pairs L1-1 and L1-3 are indicated above the L1 sequence.

Supplementary Figure S2. Tests of the L1 suppression potency of TREX1 or its mutants in a neomycin-based L1 retrotransposition assay. (**A**) TREX1 WT and mutants potently suppress L1 activity in a L1 retrotransposition assay using neomycin-resistance as the reporter. 500 ng of empty vector or the TREX1 expression plasmids (TREX1-WT or mutants: D130A, R114H, D200N) were co-transfected with 2 μg LcRPS-mneoI (L1 neo) into HeLa-HA cells seeded on a 12-well plate. The cells were subjected to G418 selection (600 μg/ml) for 13 days, and then were visualized by 0.4% Giemsa staining. (**B**) The bar chart shows the average G418-resistant colony counts of each sample. The western blotting results show the expressed levels of TREX1 and its mutants. All the data shown in this figure are representative of at least three independent experiments. The error bars shown in **B** indicate the S.D. of three replicates within one experiment.

Supplementary Figure S3. Location of specific residues in TREX1. The structure of TREX1 has been previously published (PDB:2OA8) (33) and was re-constructed with PyMol software (version 1.6.9). A monomer of TREX1 binding with DNA substrate is shown. The protein is labeled in green, with exonuclease-associated positions in red and AGS-associated positions in blue.

Supplementary Figure S4. Both DNase activity and ORF1p-degradation activity of TREX1 play important roles in reducing retroelemental L1 DNA in the cytoplasm. (**A**) qRT-PCR results of endogenous levels of L1 DNA in the cytoplasm of HeLa cells expressing exogenous TREX1 or its mutants. 1 μg VR1012 empty vector or TREX1 expression plasmids (TREX1-WT or mutants: D130A, R114H, D200N) were transfected into HeLa cells seeded on a 12-well plate. At four days post-transfection, the transfected cells were harvested and processed for cytoplasm-nuclei separation to obtain the cytoplasmic content. The cytoplasmic total DNA was then extracted and tested with L1-specific primers (the primers were named as L1-1 and L1-3) in qRT-PCR. The p-value for primer L1-3 is the same as the p-value for primer L1-1 as shown. (**B**) The western blotting results showing the expressed levels of TREX1 and its mutants. (**C**) The western blotting results show the efficiency of cytoplasm-nuclei separation. α-Tubulin serves as a protein marker for the cytoplasm part, while H3-histone for the nuclei part. All the data shown in this figure are representative of at least three independent experiments. The error bars shown in **A** indicate the S.D. of three replicates within one experiment.

Supplementary Figure S5. Exonuclease-deactivated mutations do not affect TREX1's potency with regard to ORF1p depletion or L1 suppression. (**A**) Western blotting results indicating that TREX1 mutants that are defective in DNA digestion share similar potency in ORF1p depletion. Vectors expressing TREX1 or its DNase-defective mutants in a dose manner (50, 150, and 450 ng) were co-transfected with 150 ng ORF1p-myc into HEK293T cells seeded on a 12-well plate. At 48 h post transfection, the cells were harvested for western blotting. (**B**) Transfection of TREX1 at different doses indicates similar anti-L1 potency for wild-type TREX1 and exonuclease-inactive mutants. Vectors expressing TREX1 or its DNase-defective mutants in a dose manner (50, 150, and 450 ng) were co-transfected with 2 μg L1-RP into HEK293T cells seeded on a 12-well plate to examine the possible potency against L1 retrotransposition. At four days post-transfection, EGFP-positive cells were determined by flow cytometry. JM111 was used as the negative control for flow cytometry gating, and VR1012 was the empty vector used as the negative control for TREX1 expression. The bar charts show L1 activity based on EGFP-positive cells detected by flow cytometry, and

the western blot above shows the protein levels of TREX1 for each sample. All the data shown in this figure are representative of three independent experiments. The error bars shown in **B** indicate the S.D. of three replicates within one experiment.

Supplementary Figure S6. TREX1 and its exonuclease-deactivated TREX1 mutants suppressing L1-induced genome damage. (**A**) Comet assay results concerning TREX1 and its exonuclease-deactivated TREX1 mutants in suppression of L1-induced genome damage. HeLa cells were transfected with L1-1FH (2 μg), along with 500 ng control vector or the vector expressing TREX1 or one of its mutants on a 12-well plate. The cells were then subjected to the comet assay and fluorescent imaging at 96 h post-transfection. (**B**) The tail moments of the comets in **A** were analyzed for 100 cells for each sample using Comet Assay IV software. (**C**) Western blotting results indicating corresponding levels of TREX1 in each sample shown in **A**. All the data shown in this figure are representative of at least three independent experiments. Three random areas are shown for each sample from the same experiment in **A**. The error bars shown in **B** indicate the S.E.M. of three independent experiments.

Supplementary Figure S7. TREX1 suppresses L1 activity by reducing ORF1p in a post-translational manner. (**A**) Cytotoxicity tests indicating that TREX1 is not toxic to HEK293T cells. Vectors expressing TREX1 in a dose manner (50, 150, and 450 ng) were transfected into HEK293T cells seeded on a 12-well plate. At 24 h post-transfection, the cells were re-seeded onto a 96-well plate. At 48 h or 96 h post-transfection, the cell survival activity was measured. DMEM medium (containing 10% FBS) was used as a negative control. (**B**) Luciferase activity results showing that neither TREX1 nor SAMHD1 affects the promoter potency of the CMV promoter or L1 5' UTR. TREX1 (500 ng) or SAMHD1 (500 ng) expression plasmids were co-tranfected with CMV-luciferase (500 ng) or L1 5'-UTR-luciferase (500 ng) expression plasmids into HEK293T cells seeded on a 12-well plate. At 48 h post-transfection, the cells were harvested for western blotting and luciferase assay. The western blotting results indicate the expression levels of TREX1 and SAMHD1. (**C**) qRT-PCR results suggesting that TREX1 does not destabilize endogenous L1 RNA. VR1012 (500 ng) empty vector or TREX1 (500 ng) or SAMHD1 (500 ng) expression plasmids were transfected into HEK293T cells seeded on a 12-well plate. At 48 h post-transfection, the endogenous L1 mRNA level was

tested with L1-specific primers (the primers were named as L1-1 and L1-3) in qRT-PCR. The western blotting results indicate the expression levels of TREX1 and SAMHD1. (**D**) The location of the specific primers (L1-3-F/ EGFP-F targeting L1-RP) used in the PCR for the assay in **E**. (**E**) PCR results suggesting that TREX1 does not destabilize full-length L1 RNA. 450 ng VR1012 empty vector or TREX1 (in a dose manner: 50, 150, and 450 ng) expression plasmids were co-transfected with L1-RP (2 μg) into HEK293T cells seeded on a 12-well plate. At 48 h post-transfection, levels of L1 mRNA transcribed from L1-RP were tested in PCR with specific primers indicated in **D**. RT- control (i.e., without reverse transcriptase) was prepared during the cDNA synthesis, and used to detect any possible DNA contamination. (**F**) The western blotting results indicate the expression levels of TREX1. (**G**) Western blot indicating that TREX1 is potent in reducing ORF1p expressed from vrORF1, a vector containing only a myc-tagged ORF1p open reading frame instead of an intact L1 sequence. ORF1-myc (150 ng) and/or TREX1 (500 ng) expression plasmids were transfected into HEK293T cells seeded on a 12-well plate. All the data shown in this figure are representative of at least three independent experiments, and the error bars in **A-C** indicate the S.D. of three replicates within one experiment.

Supplementary Figure S8. TREX1 reduces the retrotransposition potency of a synthetic human L1. (**A**) Cartoon showing the DNA differences between endogenous L1, L1-RP, and the synthetic human ORFeus-HS (sL1). Different from L1-RP, the ORF1 and ORF2 in sL1 have been codon-optimized. In addition, a CMV promoter followed by a Kozak sequence (K) replaces the original 5’ UTR in L1-RP. (**B**) L1 assay results showing that TREX1 has potency against L1-RP and sL1 activity that is similar to that of SAMHD1, a protein which reduces ORF2p levels (11) and promotes stress granule formation by ORF1p (60). VR1012 (500 ng) empty vector or TREX1 (500 ng) or SAMHD1 (500 ng) expression plasmids were co-transfected with L1-RP (2 μg) or sL1 (1 μg) into HEK293T cells seeded on a 12-well plate. At four days post-transfection, EGFP-positive cells were determined by flow cytometry. The western blotting results on the right indicate the expression levels of TREX1 and SAMHD1. All the data shown in this figure are representative of at least three independent experiments, and the error bars in **B** indicate the S.D. of three replicates within one experiment.

Supplementary Figure S9. Endogenous TREX1 expression in HeLa and HEK293T cells. Western blotting results indicate that TREX1 is potently expressed in HeLa cells but not HEK293T cells.

Supplementary Figure S10. Fixation of cells does not alter the co-localization of TREX1 and ORF1p. HEK293T cells were transfected with ORF1p-mCherry (red, 1 μg) and/or TREX1-GFP (green, 1 μg) expression vectors on a 6-well plate. The cells were fixed with paraformaldehyde at 24 h post-transfection and subjected to fluorescence imaging. The pictures shown were taken from one experiment and are representative of three independent experiments.

Supplementary Figure S11. TREX1 induces ORF1p removal through proteasome proteolysis. (A) Western blotting results showing that the proteasome inhibitor MG132 potently rescues APOBEC3G from Vif-induced degradation. VR1012 (900 ng) empty vector or Vif (900 ng) expression plasmid was co-transfected with A3G (300 ng) into HEK293T cells seeded on a 12-well plate. (**B**) Western blotting results indicating that the proteasome inhibitor MG132 rescues ORF1p from TREX1-mediated depletion. VR1012 (500 ng) empty vector or TREX1 (500 ng) expression plasmid was co-transfected with L1-1FH (300 ng) into HEK293T cells seeded on a 12-well plate. All the data shown in this figure are representative of at least three independent experiments.

Supplementary information references

60. Hu, S., Li, J., Xu, F., Mei, S., Le Duff, Y., Yin, L., Pang, X., Cen, S., Jin, Q., Liang, C. *et al.* (2015) SAMHD1 Inhibits LINE-1 Retrotransposition by Promoting Stress Granule Formation. *PLoS Genet*, **11**, e1005367.

Supplementary Figure S1

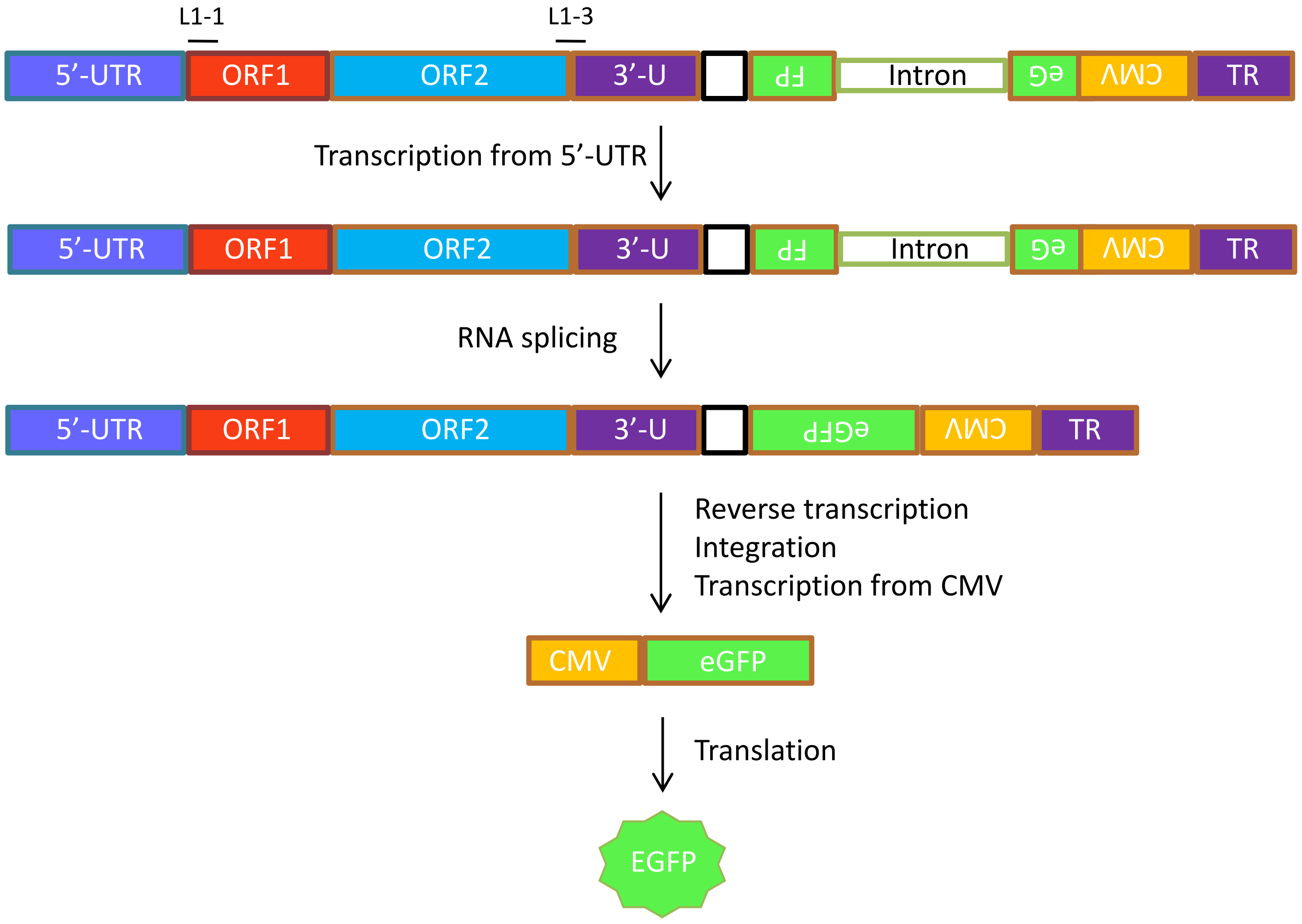

Supplementary Figure S2

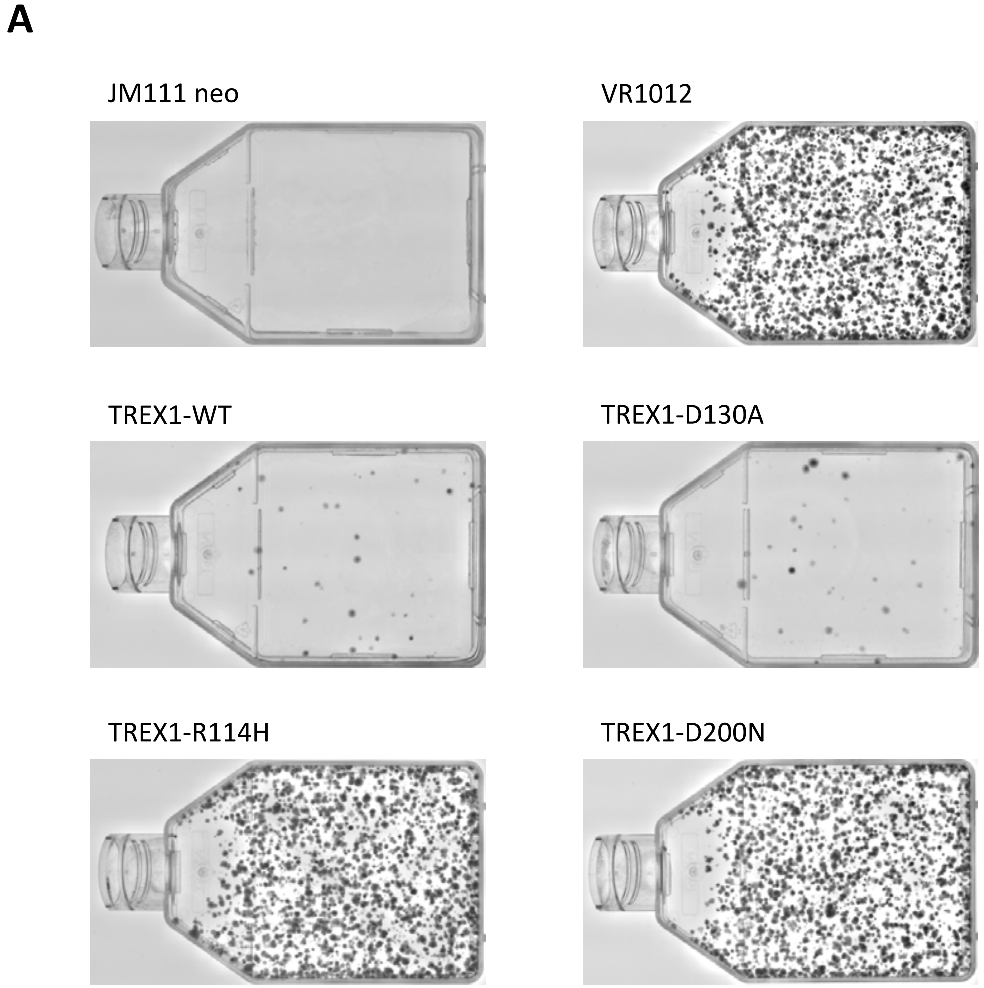

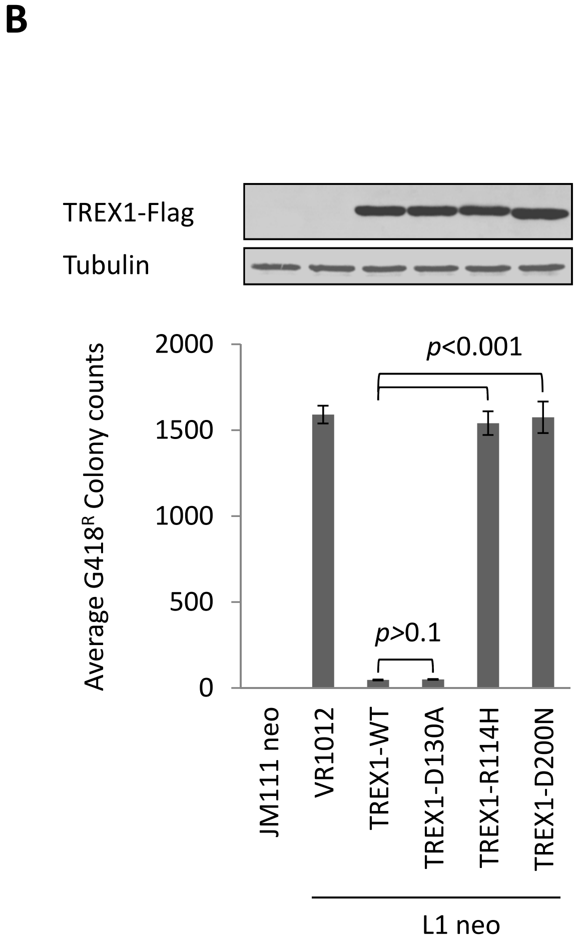

Supplementary Figure S3

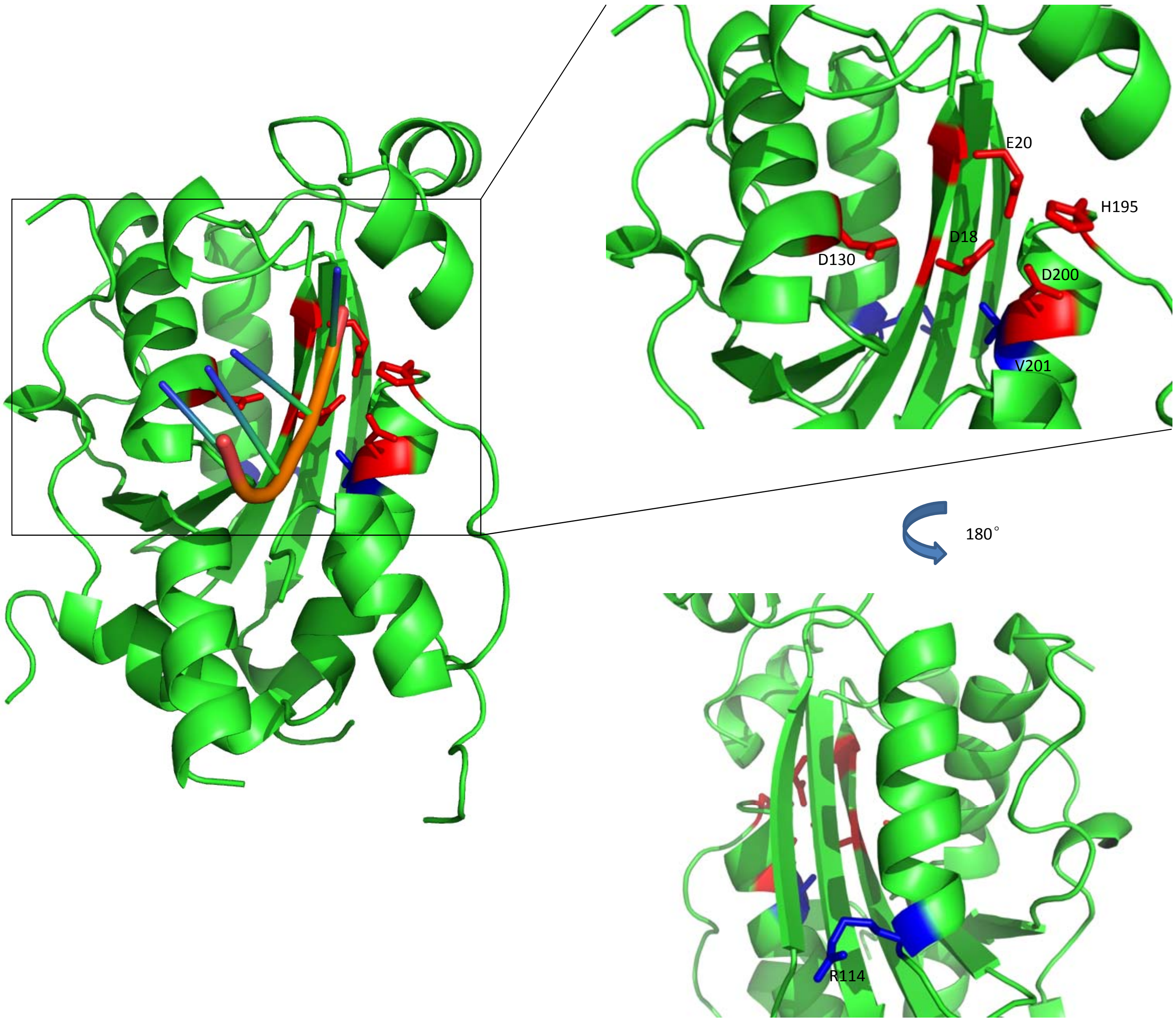

Supplementary Figure S4

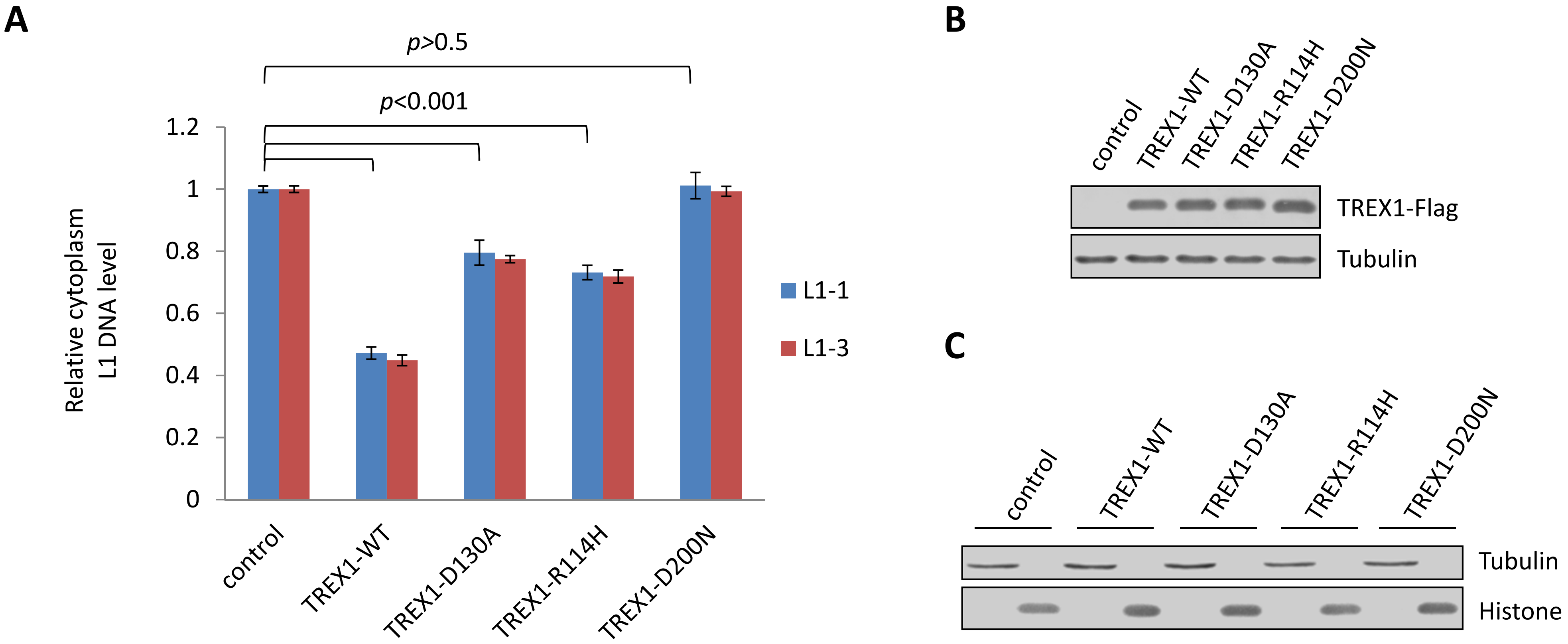

Supplementary Figure S5

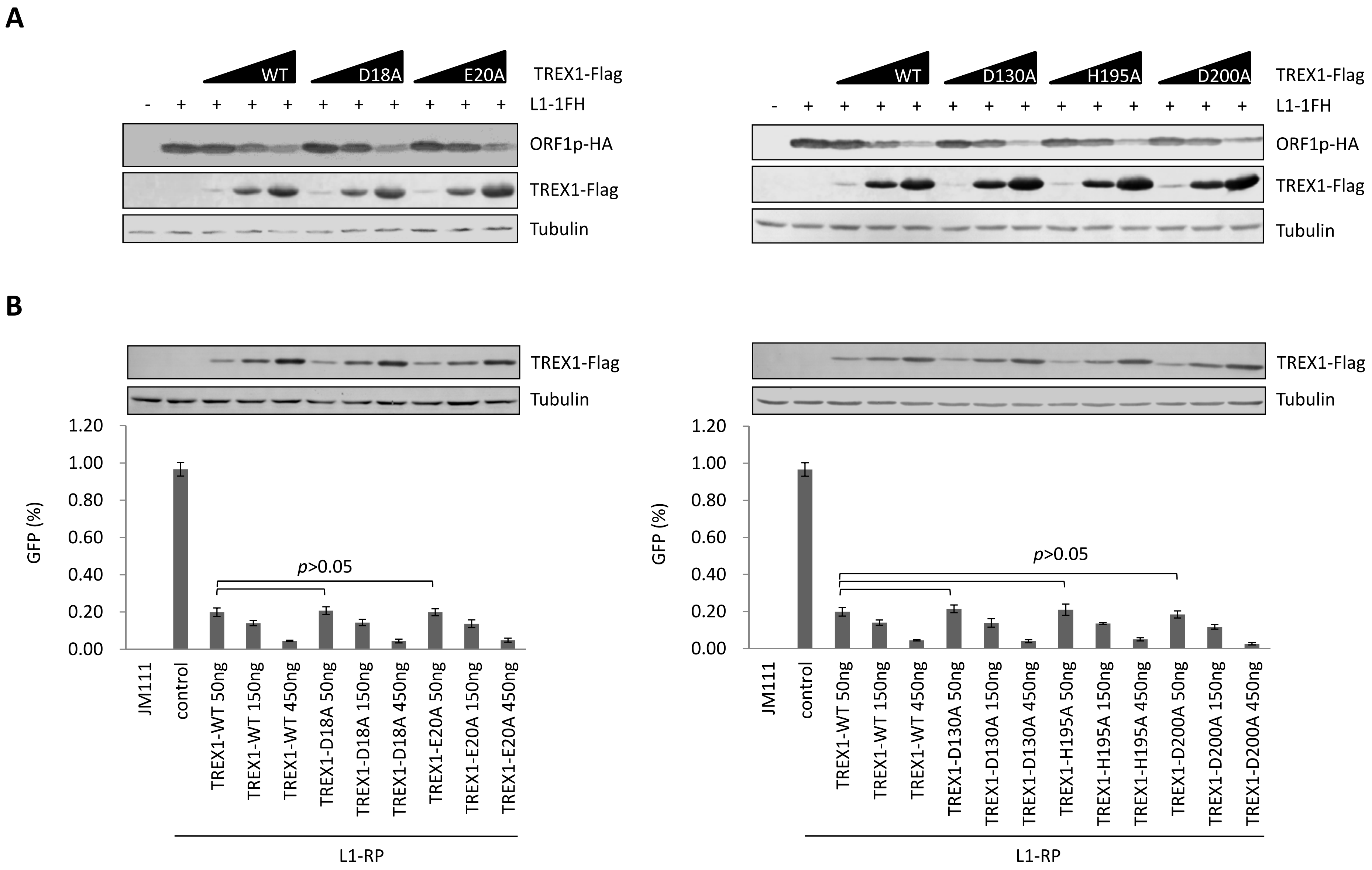

Supplementary Figure S6

A

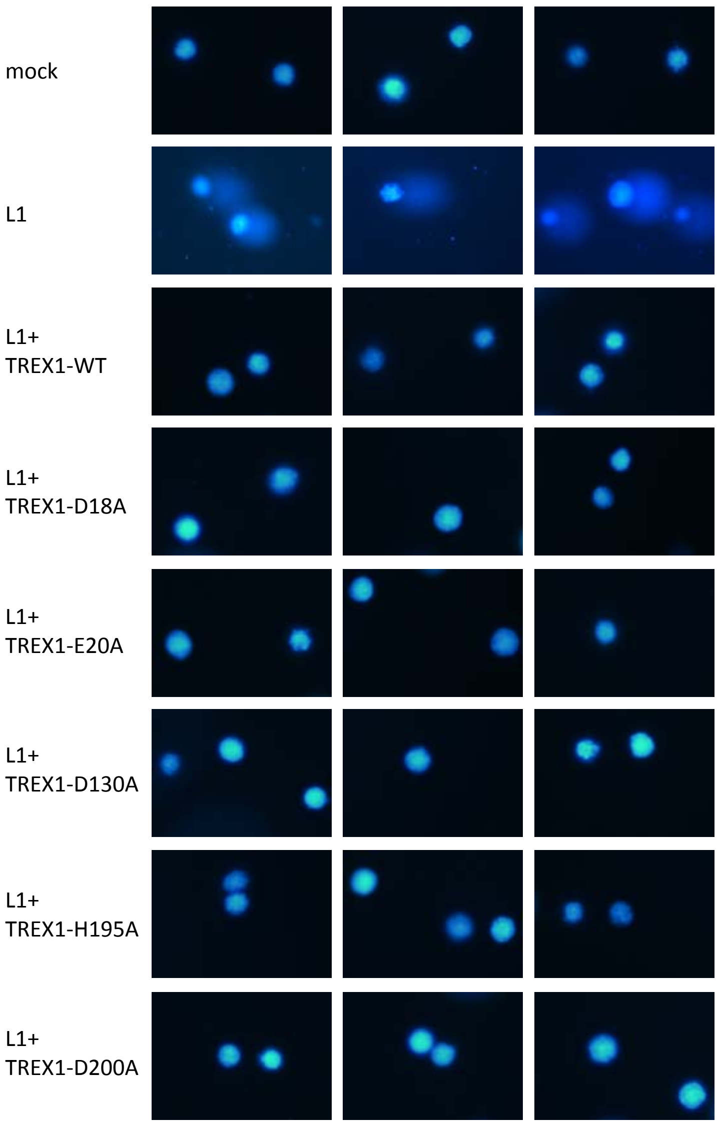

B

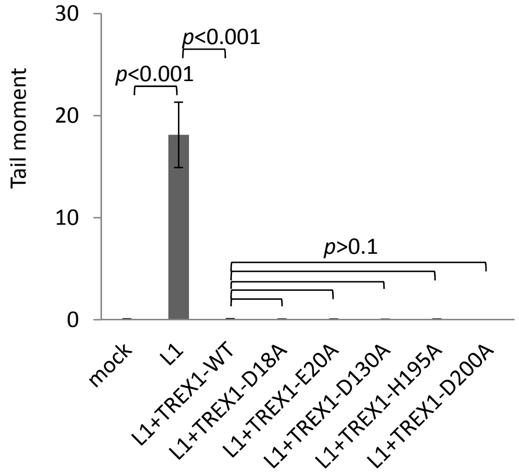

C

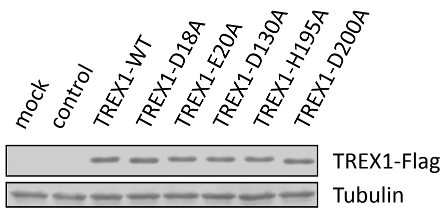

Supplementary Figure S7

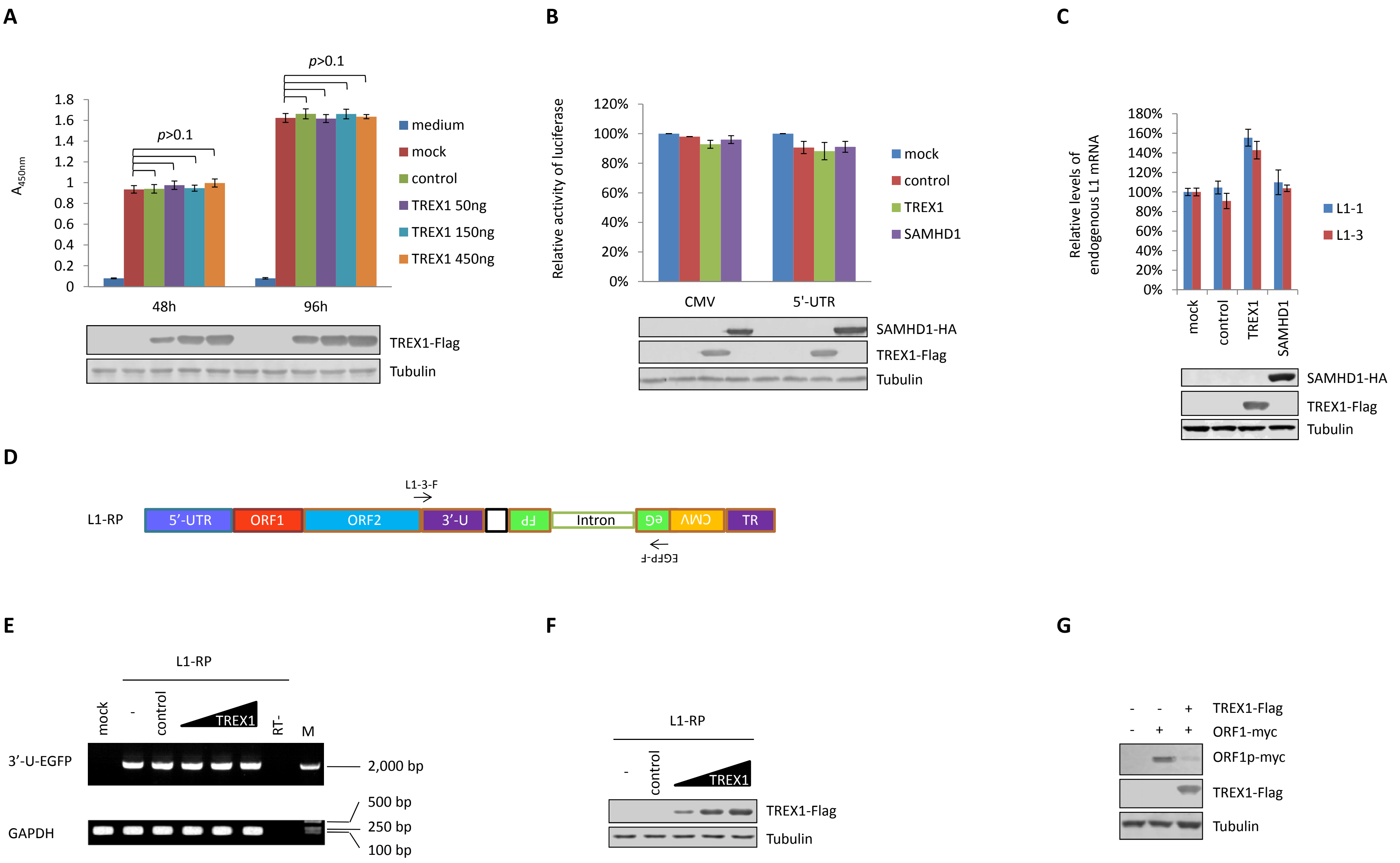

Supplementary Figure S8

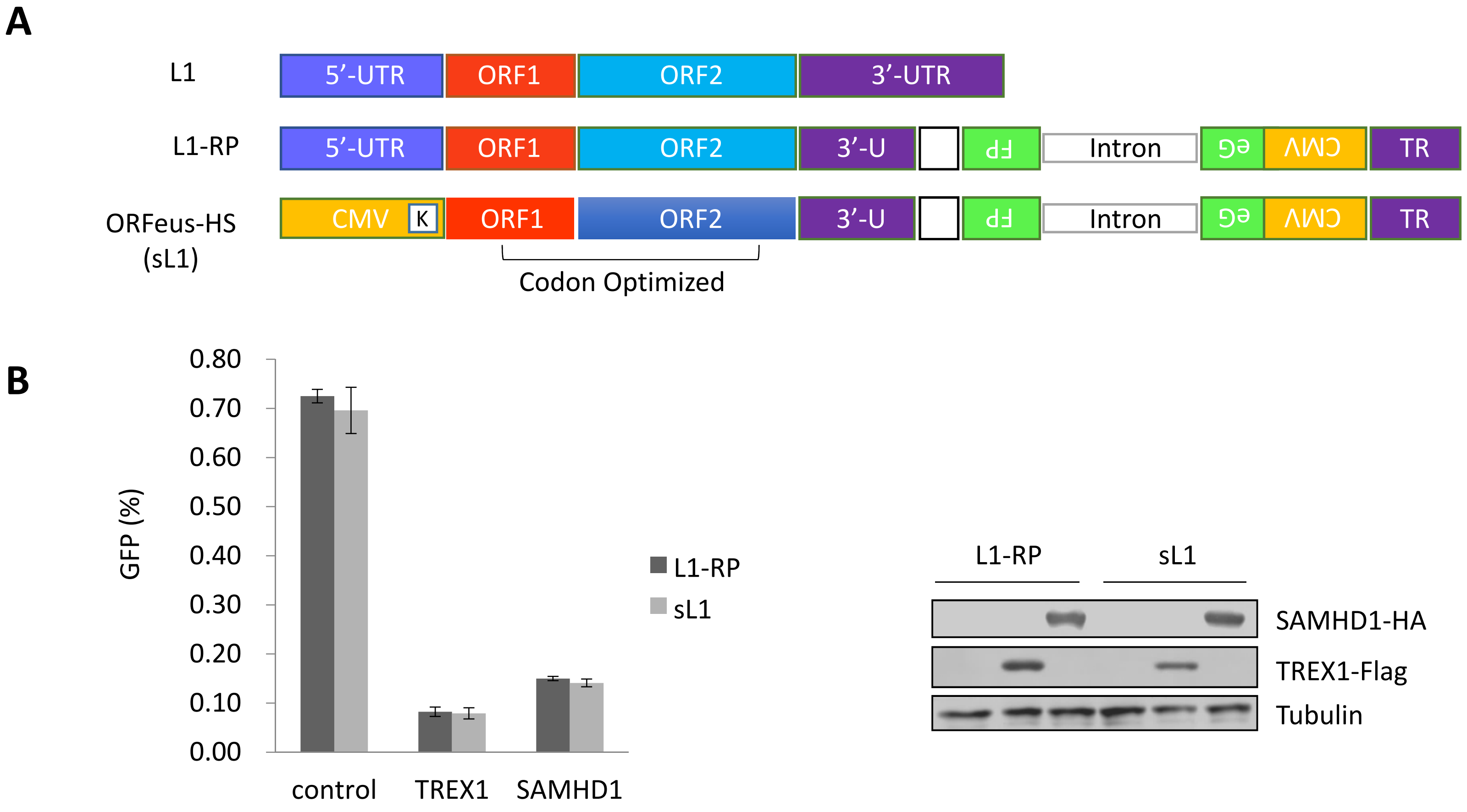

Supplementary Figure S9

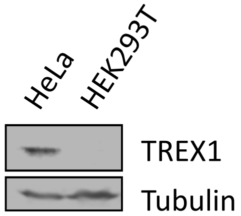

Supplementary Figure S10

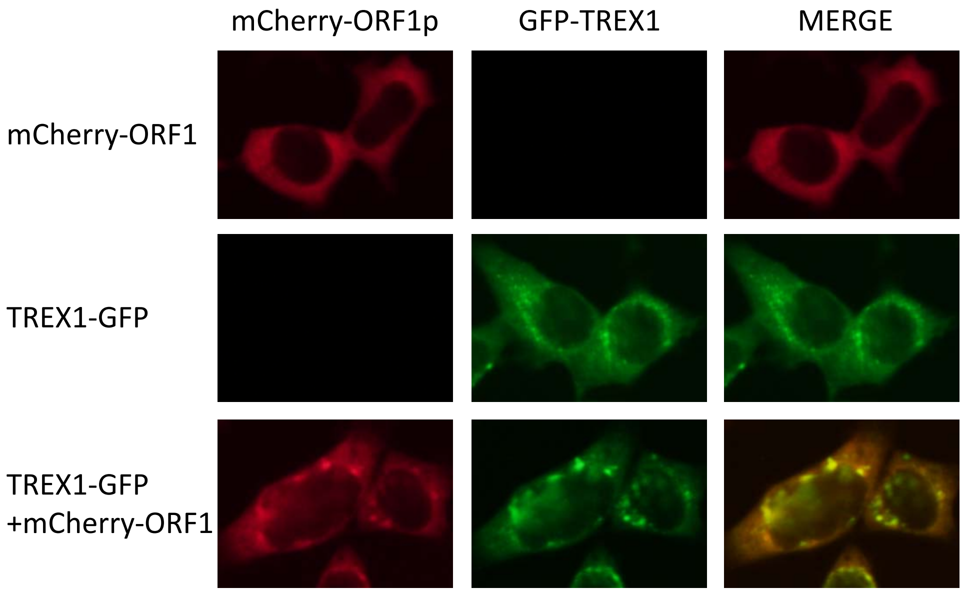

Supplementary Figure S11

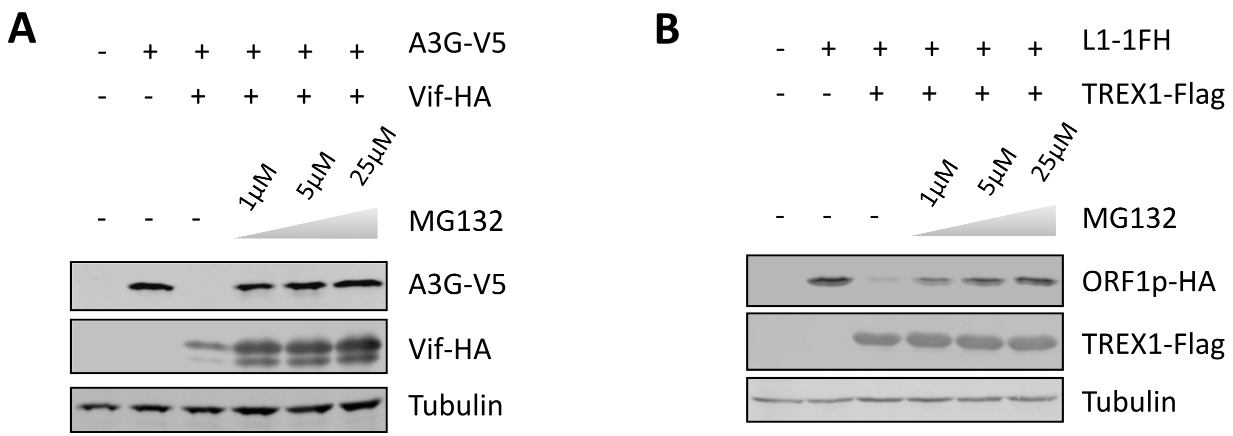

Supplementary Table 1. Description of reference genes and parameters derived from qRT-PCR.

Genes	Gene Function	Gene Accession Number	Primer Sequence (Forward/Reverse)	Tm (°C)	Product (bp)	Amplification Efficiency (%)	R^2
ACTB	structural constituent of cytoskeleton	E00829.1	ACCGAGCGCGGCTACAG/ CTTAATGTCACGCACGATTTCC	60°C	60bp	99.2	0.994
L1	human retroelement	L19088.1	L1-1: GAATGATTTTGACGAGCTGAGAGAA/ GTCCTCCCGTAGCTCAGAGTAATT	60°C	67bp	99.4	0.996
L1	human retroelement	L19088.1	L1-3: CAAACACCGCATATTCTCACTCA/ CTTCCTGTGTCCATGTGATCTCA	60°C	64bp	100.6	0.997
